# Latent Chronic Osteomyelitis Presenting Decades After Initial Trauma: A Case Report and Literature Review

**DOI:** 10.7759/cureus.61789

**Published:** 2024-06-06

**Authors:** Obada Alalman, Georges Sakhat, Elie Alam, Hassan Mallat, Mario Chalouhi

**Affiliations:** 1 Orthopedic Surgery, Lebanese University, Beirut, LBN; 2 Orthopedic Surgery, University of Balamand, Beirut, LBN; 3 Infectious Diseases, Doctoral School of Sciences and Technology, Lebanese University, Tripoli, LBN

**Keywords:** marjolijn’s ulcer, recurrence, bone biopsy, staphylococcus aureus, osteomyelitis

## Abstract

Osteomyelitis is an inflammatory bone process secondary to infection, and often presents as a chronic, recurrent illness, posing diagnostic and treatment challenges. It is frequently the result of previous inadequate treatment or undiagnosed acute infection. Clinical suspicion, thorough evaluation, laboratory studies, and advanced imaging modalities such as magnetic resonance imaging (MRI) and computed tomography (CT) play crucial roles in diagnosis. Treatment typically entails a combination of antibiotics and surgical debridement to eliminate infection and facilitate bone healing.

In this report, we present a rare case involving a 64-year-old male who presented with new-onset pain in the right femur decades after experiencing a complicated femur fracture and forearm crush injury. Imaging studies revealed evidence of chronic osteomyelitis, leading to a diagnosis of latent infection. The patient underwent a comprehensive evaluation, including clinical examination, imaging studies, laboratory tests, and bone biopsy, confirming the diagnosis. This case of latent osteomyelitis highlights the clinical presentation, diagnostic modalities, and treatment strategies employed in managing this challenging condition

## Introduction

Osteomyelitis is an acute or chronic bone inflammatory process secondary to infection by pyogenic organisms, including bacteria, fungi, and mycobacteria [1]. Chronic osteomyelitis may present as a recurrent illness. The symptoms and their duration may vary considerably, whereas dormancy periods can also be of variable duration [2].

Chronic silent osteomyelitis typically arises from a previous episode of acute osteomyelitis that is inadequately treated or not promptly diagnosed [3]. Bacterial pathogens, particularly *Staphylococcus aureus*, are the most common causative agents, accounting for most cases [3,4]. These pathogens gain access to the bone through various routes, including direct inoculation, hematogenous spread, or contiguous spread from adjacent infected sites [4].

Several factors increase the risk of chronic silent osteomyelitis, including immune compromise and chronic diseases, for example, diabetes, by impairing the immune system, compromising blood flow to the affected area, and favoring chronic silent osteomyelitis [4].

As the infection progresses, more specific symptoms may manifest, most commonly persistent pain in the affected area that can be attributed to ongoing bony inflammation [5]. It is crucial for individuals experiencing chronic fatigue, malaise, unexplained fever, persistent pain, limited range of motion, or bone deformities to seek medical advice and discuss these symptoms with healthcare professionals [6]. Unlike acute osteomyelitis, which presents as localized pain, swelling, and redness, individuals with chronic silent osteomyelitis often have nonspecific complaints that can easily be overlooked or attributed to other causes [4,7].

Healthcare providers employ various diagnostic modalities to overcome the diagnostic challenges of osteomyelitis. These include clinical evaluation, imaging studies, laboratory tests, and microbiological cultures [7]. Clinical evaluation involves a thorough physical examination to assess signs of infection, such as localized tenderness, warmth, or swelling [4].

Plain radiographs are often the initial imaging modality used and can reveal bony changes associated with chronic osteomyelitis: bone destruction, periosteal reaction, and sequestrum formation [4,7]. However, early-stage osteomyelitis may not be apparent on plain radiographs, especially without significant bony changes. In such cases, more advanced imaging techniques such as magnetic resonance imaging (MRI) and computed tomography (CT) may be necessary [8]. Laboratory tests are not specific to osteomyelitis and can be elevated in other inflammatory conditions but can support the diagnosis of osteomyelitis. Elevated white blood cell (WBC) count, erythrocyte sedimentation rate (ESR), and C-reactive protein (CRP) levels indicate an ongoing inflammatory process [7]. Microbiological cultures are important for diagnosis and treatment; they can be obtained through aspiration or biopsy of the affected bone or surrounding soft tissues [7].

Managing bone infections poses significant challenges in clinical practice, and untreated or inadequately managed chronic silent osteomyelitis can lead to severe complications [9]. One of the primary consequences is the progressive destruction of bone tissue, which can result in structural deformities and compromised skeletal integrity. This bone destruction can extend beyond the affected site and may lead to joint instability, impairing mobility and function [2,9].

## Case presentation

A 64-year-old male, with no known chronic medical illnesses or allergies, was previously operated on for a right femoral diaphyseal fracture around five decades ago (in 1975) with an intramedullary femoral nail, following a motor vehicle accident (MVA). At the time of the MVA, he also sustained a right forearm fracture, which was fixated with IM pinning. His postoperative recovery was complicated by two separate skin and soft tissue infections. The first occurred in his right forearm six months after initial treatment, which required hospital admission for IV antibiotics and removal of hardware. The second infection occurred one year after the MVA in his right thigh. He was admitted for IV antibiotics and hardware removal, then discharged home on a course of intramuscular antibiotics (The patient does not recall the name of the antibiotic and his previous medical files, and the imaging studies were inaccessible.). The patient denied any recurrence of his symptoms or any complications since then.

Seven months prior to his presentation in 2022, he started experiencing needle-like pain in his right thigh, radiating to the right calf and shin, which became progressively more severe during the last three months. Pain reported had a nocturnal pattern, associated with hotness in the right thigh, pruritus, and night sweats. No fever, chills, or other symptoms were reported. The pain would be alleviated with the use of oral acetaminophen and activity.

**Figure 1:**
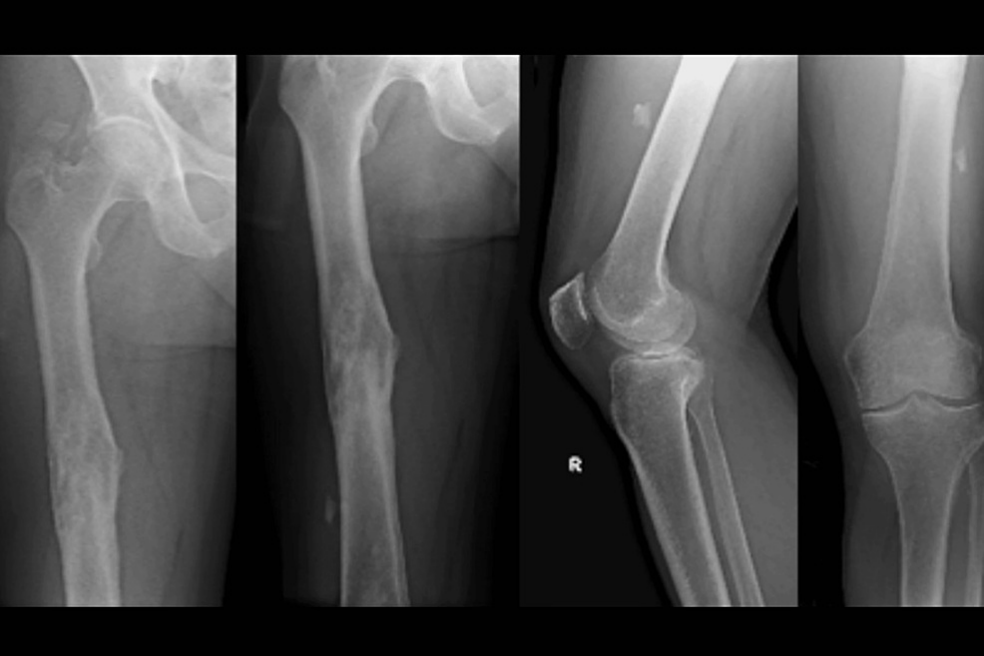
Preoperative radiographs of the right femur, and the surrounding hip and knee joints show bony changes in the femoral mid-shaft.

**Figure 2:**
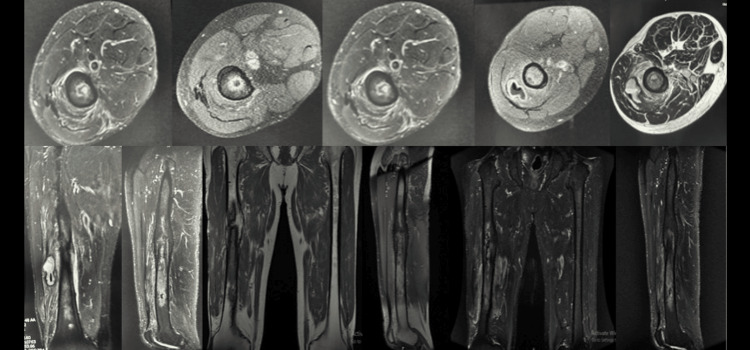
MRI of the thighs with axial, coronal, and sagittal views shows a 28×10 mm intraosseous formation with peripheral contrast enhancement consistent with an abscess, also seen in STIR views. MRI, magnetic resonance imaging

Lab workup, radiographs, and an MRI were performed. Radiographs (Figure 1) showed bony changes, such as periosteal thickening with loss of trabecular architecture. MRI (Figure 2) demonstrated diaphyseal edema and an intraosseous formation with contrast enhancement in the distal femoral diaphysis, 11 cm proximal to the femoral condyles, measuring 28x10 mm in the sagittal plane, compatible with an intraosseous abscess (Figure 2). The MRI was also notable for an intramuscular abscess of the vastus intermedius measuring 5.5x2.8 cm in the sagittal plane, located 13 cm proximal to the femoral condyles. He was diagnosed with chronic osteomyelitis of the right femur (Figures 1, 2).

His laboratory workup revealed a WBC count of 8.81 x 10^3^/mL (normal values of 4.4-11.3) with 60.4% neutrophils as well as an ESR of 77 mm/h (normal values of 0-20.0) and a CRP of 83.0 mg/L (normal values of 0-5.0).

**Figure 3:**
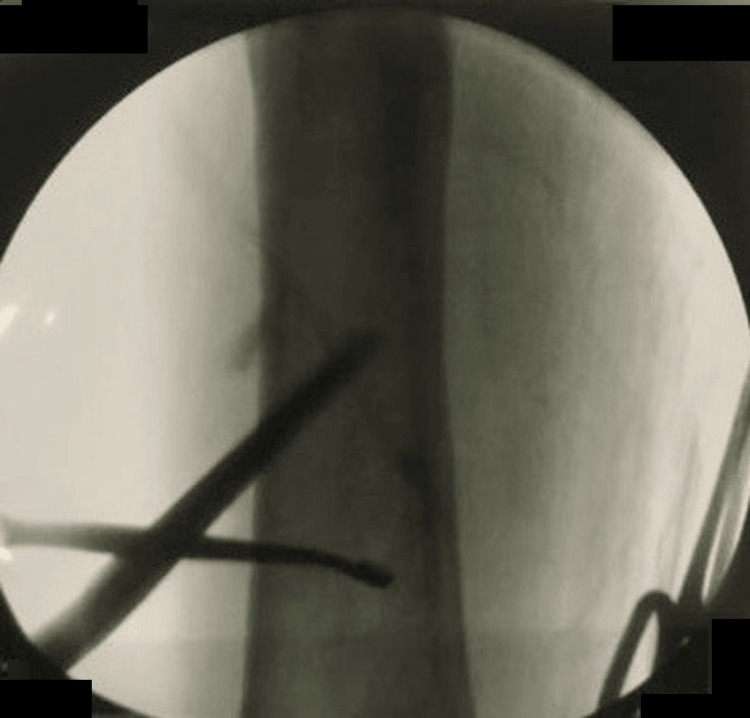
Intraoperative fluoroscopy image of the right femur demonstrates the opening of the cortical window using an awl to access the bone abscess in the intramedullary canal for debridement, drainage, cultures, and biopsy.

**Figure 4:**
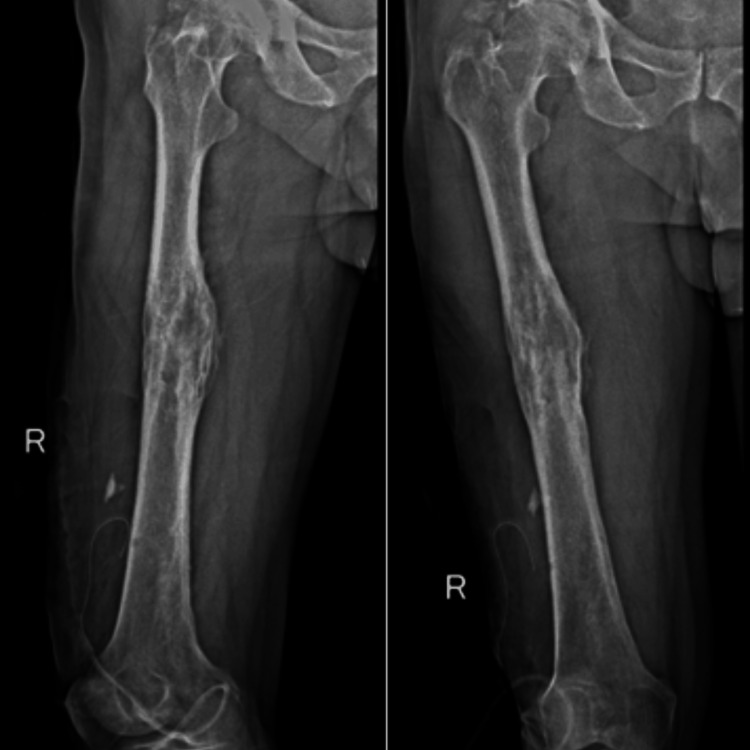
Postoperative radiographs of the right femur show the intraoperatively inserted drain.

The patient was admitted for surgical treatment. He underwent drainage of the intramuscular and intraosseous abscesses by opening a cortical window (Figures 3, 4). Samples were taken for cultures and biopsies. A drain was placed (Figure 4) and subsequently removed four days after the surgery. The patient was started immediately postoperatively on IV antibiotics: he initially received a course of vancomycin 1 g IV every 12 hours with cefepime 1 g IV every eight hours empirically; intraoperative cultures revealed growth of *S. aureus*, which was sensitive to methicillin (MSSA) and ciprofloxacin, as such he was switched by the hospital’s infectious diseases team to ciprofloxacin 400 mg IV every 12 hours with ceftriaxone 2 g IV every 24 hours. The patient had a smooth postoperative course and was discharged on postoperative day six. Ciprofloxacin (500 mg PO twice daily) was prescribed for six weeks.

Bi-monthly lab tests revealed a gradual decrease in his CRP count, reaching 7.7 mg/L at six weeks postoperatively. At two months postoperative, he experienced mild right thigh pain associated with an increase of his CRP to 32.4 mg/L; he was subsequently started on a course of cefadroxil 1 g PO twice daily for two weeks. His CRP count normalized after two weeks with the resolution of his pain.

At one and a half years postoperative follow-up, the patient reports being symptom-free, with a full return to function noted. A physical exam showed a clean surgical wound and no residual tenderness to palpation.

## Discussion

Chronic silent osteomyelitis is a well-known but rare complication after a complex fracture and previous osteomyelitis, and few cases were reported in the literature, including a case of late infection 30 years after a healed complex fracture [10].

The relapse rates following bony infection have been reported to be as high as 20%-30%, making its management more challenging [11,12]. Chronic osteomyelitis appears as a recurrent or intermittent disease. The clinical signs and symptoms may vary considerably, whereas latency periods can also be of variable duration [2]. Among various types of osteomyelitis, Methicillin-sensitive *S. aureus* is consistently the predominant pathogen, followed by* Pseudomonas aeruginosa* and methicillin-resistant *S. aureus* in frequency of identification [4]. Following the introduction of the pathogen into the intramedullary cavity, it adheres to membrane proteins such as fibronectin or collagen receptors, establishing an infection [13]. For example, *S. aureus* has been reported to survive within host cells, surrounded by a biofilm; such pathogens present with an altered phenotype concerning growth, gene expression, and protein production that protects them from the host’s defense mechanisms as well as the systemic effect of antibiotics [3,13,14]. The pathogens can remain in this dormancy state for a long period and may cause flare-ups many years after the initial inoculation [5].

An early diagnosis of chronic osteomyelitis would lead to a more favorable outcome and fast recovery, decreasing the complications and the recurrence rates. The diagnosis of chronic osteomyelitis can often be challenging, therefore a combination of a high index of clinical suspicion based on a detailed history and clinical symptoms, along with imaging and laboratory investigations, can help in the diagnosis [2]. The clinical signs of chronic osteomyelitis are usually non-specific and are therefore difficult to recognize; it can also be difficult to differentiate the signs of osteomyelitis from soft tissue infections, especially in patients with diabetes. A variety of symptoms have been reported, ranging from no skin lesions to open wounds over fractured bones [2].

Imaging plays a crucial role for physicians, aiding in the diagnosis and treatment planning of osteomyelitis, starting with plain radiographs, as they exclude other diagnostic possibilities and provide clues for underlying pathologic conditions. Plain radiographs may show bony and soft tissue changes like muscle swelling [8]. Cross-sectional imaging modalities, such as CT and MRI, are now considered standard for diagnosing osteomyelitis due to their high sensitivity and specificity. These modalities provide excellent anatomic delineation of the infected area and surrounding soft tissues. In contrast, nuclear medicine techniques are sensitive but sometimes nonspecific [8]. Although MRI is of limited value in the presence of implants, scar tissue, and recent operations, it is considered the most useful imaging technique to evaluate suspected osteomyelitis because of its ability to demonstrate changes in the water content of bone marrow with an excellent structural definition and spatial resolution [15]. MRI evaluates the bone and its adjacent soft tissues, identifying early signs such as edema and hyperemia [16]. Moreover, it assists in treatment planning by estimating the necessary margins for debridement and evaluating therapy response [16].

Routine laboratory tests lack specificity when not supported with other radiologic and microbiologic data for osteomyelitis diagnosis. The WBC count could be normal, and the ESR and CRP are often elevated; In cases of proven osteomyelitis, those tests may be used to evaluate response to therapy or relapse [17].

The gold standard to diagnose osteomyelitis is bone biopsy with histopathologic examination and tissue culture, the presence of positive microbial cultures from bone tissues around areas of bone necrosis is considered essential for diagnosis and treatment planning [6]. Specimens should undergo both aerobic and anaerobic bacterial cultures. In addition, fungal and mycobacterial cultures should be performed if suspected [18].

The treatment aims to eliminate the inflammatory process; it can be achieved by the combination of antibiotics and surgical excision and debridement by removing all the pathogens in the devitalized tissues and sequestra (dead bone), to reach healthy and viable tissues, promoting healing by optimizing the mechanical and biological environment and decreasing the recurrence rates [2,13]. Initial empiric antibiotic treatment should be started as soon as the culture samples have been obtained. Then, it should be adjusted according to the results of the cultures and the microorganism's susceptibility to antibiotics [2]. Following debridement, specimens should be collected from the affected bone, and its surrounding soft tissues for pathological examination to rule out malignant transformations [19]. The literature indicates that oral administration is comparable in effectiveness to parenteral administration, with fewer adverse reactions observed in patients undergoing oral therapy [18]. Furthermore, a systematic review by Conterno et al. showed no difference between oral antibiotics when compared with parenteral antibiotics in the rate of remission at the end of therapy and after 12 months or more of follow-up [13]. There is no significant difference between short-term and long-term antimicrobial treatment for osteomyelitis, according to randomized controlled trials (RCTs) and meta-analyses; nevertheless, the evidence is limited and indicates that high-risk patients might need at least six weeks of antibiotic treatment [18].

Many reported complications may arise due to chronic inflammation and infective processes, such as abscess formation and sinus tracts and extension to adjacent tissues are considered the most common complications [2]. The most important, but rare and long-term complication, in chronic osteomyelitis is the malignant transformation, called Marjolijn’s ulcer. It can manifest at any point during the illness, typically occurring after a decade or longer. Early diagnosis and histopathological examination are crucial for appropriate surgical treatment [20].

## Conclusions

We present a case of latent osteomyelitis of the right femur, emerging approximately 50 years after complex femur and forearm fractures that had been complicated by osteomyelitis. We considered that the osteomyelitis remained dormant for this long period.

Chronic silent osteomyelitis should always be considered in cases presenting with pain at the site of previous trauma and infection, exhibiting unusual characteristics such as nocturnal, new-onset chronic, radiating pain, as well as other atypical and systemic symptoms. Early diagnosis using various imaging modalities, laboratory studies, cultures, and biopsies can be challenging but is essential to prevent further bone and soft tissue destruction and other serious complications and to plan appropriate treatment. Treatment typically involves a combination of antibiotics and extensive surgical excision, tailored to each case, to achieve early recovery and prevent recurrence.

This case underscores the importance of remaining vigilant in clinical evaluation and considering the possibility of chronic latent osteomyelitis, particularly in patients with a history of trauma and infection, even after an extended period.

## References

[1] Momodu II, Savaliya V (2024). Osteomyelitis.

[2] Panteli M, Giannoudis PV (2016). Chronic osteomyelitis: what the surgeon needs to know. EFORT Open Rev.

[3] Dym H, Zeidan J (2017). Microbiology of acute and chronic osteomyelitis and antibiotic treatment. Dent Clin North Am.

[4] Grundy SM, Vega GL (2022). Statin intolerance and noncompliance: an empiric approach. Am J Med.

[5] Mandell JC, Khurana B, Smith JT, Czuczman GJ, Ghazikhanian V, Smith SE (2018). Osteomyelitis of the lower extremity: pathophysiology, imaging, and classification, with an emphasis on diabetic foot infection. Emerg Radiol.

[6] Jha Y, Chaudhary K (2022). Diagnosis and treatment modalities for osteomyelitis. Cureus.

[7] Hatzenbuehler J, Pulling TJ (2011). Diagnosis and management of osteomyelitis. Am Fam Physician.

[8] Pineda C, Espinosa R, Pena A (2009). Radiographic imaging in osteomyelitis: the role of plain radiography, computed tomography, ultrasonography, magnetic resonance imaging, and scintigraphy. Semin Plast Surg.

[9] Birt MC, Anderson DW, Bruce Toby E, Wang J (2017). Osteomyelitis: recent advances in pathophysiology and therapeutic strategies. J Orthop.

[10] Ahmad SS, Kohl S, Evangelopoulos DS, Krüger A (2013). Silent chronic osteomyelitis lasting for 30 years before outburst of symptoms. BMJ Case Rep.

[11] Mathews JA, Ward J, Chapman TW, Khan UM, Kelly MB (2015). Single-stage orthoplastic reconstruction of Gustilo-Anderson Grade III open tibial fractures greatly reduces infection rates. Injury.

[12] Lazzarini L, Mader JT, Calhoun JH (2004). Osteomyelitis in long bones. J Bone Joint Surg Am.

[13] Conterno LO, Turchi MD (2013). Antibiotics for treating chronic osteomyelitis in adults. Cochrane Database Syst Rev.

[14] Panteli M, Puttaswamaiah R, Lowenberg DW, Giannoudis PV (2014). Malignant transformation in chronic osteomyelitis: recognition and principles of management. J Am Acad Orthop Surg.

[15] Meyers SP, Wiener SN (1991). Diagnosis of hematogenous pyogenic vertebral osteomyelitis by magnetic resonance imaging. Arch Intern Med.

[16] Sambri A, Spinnato P, Tedeschi S (2021). Bone and joint infections: the role of imaging in tailoring diagnosis to improve patients’ care. J Pers Med.

[17] Fritz JM, McDonald JR (2008). Osteomyelitis: approach to diagnosis and treatment. Phys Sportsmed.

[18] Besal R, Adamič P, Beović B, Papst L (2023). Systemic antimicrobial treatment of chronic osteomyelitis in adults: a narrative review. Antibiotics (Basel).

[19] Mouzopoulos G, Kanakaris NK, Kontakis G, Obakponovwe O, Townsend R, Giannoudis PV (2011). Management of bone infections in adults: the surgeon’s and microbiologist’s perspectives. Injury.

[20] Steinrücken J, Osterheld MC, Trampuz A (2012). Malignancy transformation of chronic osteomyelitis: description of 6 cases of Marjolin’s ulcers. Eur J Orthop Surg Traumatol.

